# Differential effect of hyperthermia and x-irradiation on regrowth rate and tumour-bed effect for a rat sarcoma.

**DOI:** 10.1038/bjc.1982.42

**Published:** 1982-02

**Authors:** T. E. Wheldon, E. C. Hingston

## Abstract

The regrowth rate of the rat sarcoma SSB1a was assessed, following treatment with either X-rays or hyperthermia. The growth rate of untreated tumours implanted in pre-irradiated or pre-heated normal tissue was also measured. For treatments giving similar levels of tumour regrowth delay, the regrowth of irradiated tumours was markedly slower than that of untreated tumours, whilst the regrowth rate of tumours subjected to hyperthermia did not differ from that of untreated tumours. The growth rate of untreated tumours implanted in pre-irradiated skin was also slower than that of control tumours, though the growth-rate reduction was less dramatic than for regrowth following tumour irradiation in situ. Tumours implanted in pre-heated skin grew at the same rate as control tumours. Slower regrowth in situ after X-rays than after hyperthermia would produce a larger growth delay for X-rays than for hyperthermia, for equal levels of tumour-cell kill or probability of tumour sterilization. This effect could lead to a systematic underestimation of the efficacy of hyperthermia relative to X-rays, when efficacy is assessed by growth delay.


*Br. J. Cancer* (1982) **45**, 265

### DIFFERENTIAL EFFECT OF HYPERTHERMIA AND X-IRRADIATION ON REGROWTH RATE AND TUMOUR-BED EFFECT FOR A RAT SARCOMA

T. E. WHELDON AND E. C. HINGSTON

*From the MRC Cyclotron Unit, Hammersmith Hospital, Du Cane Road, London W12 0HS*

Received 24 August 1981 Accepted 2 October 1981

**Summary.—The regrowth rate of the rat sarcoma SSB1a was assessed, following treatment with either X-rays or hyperthermia. The growth rate of untreated tumours implanted in pre-irradiated or pre-heated normal tissue was also measured.**

**For treatments giving similar levels of tumour regrowth delay, the regrowth of irradiated tumours was markedly slower than that of untreated tumours, whilst the regrowth rate of tumours subjected to hyperthermia did not differ from that of untreated tumours. The growth rate of untreated tumours implanted in pre-irradiated skin was also slower than that of control tumours, though the growth-rate reduction was less dramatic than for regrowth following tumour irradiation *in situ*. Tumours implanted in pre-heated skin grew at the same rate as control tumours.**

**Slower regrowth *in situ* after X-rays than after hyperthermia would produce a larger growth delay for X-rays than for hyperthermia, for equal levels of tumour-cell kill or probability of tumour sterilization. This effect could lead to a systematic underestimation of the efficacy of hyperthermia relative to X-rays, when efficacy is assessed by growth delay.**

In COMPARING the relative effectiveness of different treatments, using regrowth delay as the end-point, it is important to determine, if possible, the contribution made by factors other than cell survival—especially if the relative contributions may differ from one modality to another. Regrowth delay as a tumour response results from (a) the level of cell survival and (b) the rate at which the surviving cells regrow. Each of these components may be affected by treatment, and the interpretation of regrowth-delay experiments is complicated by the possibility that kinetic changes induced by the treatment play a significant role in the observed response (see Brown & Howes, 1974; McNally, 1974).

In the present report, a comparative study is made of growth changes in kinetics induced by X-rays or hyperthermia. A growth-slowing effect of irradiation is well recognized and may result, at least in part, from vascular damage to the normal tissues which constitute the tumour bed (Stenstrom *et al.*, 1955; Summers *et al.*, 1964; Thomlinson & Craddock, 1967; Hewitt & Blake, 1968). "Tumour-bed effect" is sometimes used to describe the slowed growth of tumours which have been irradiated *in situ*, as well as that of untreated tumours growing in pre-treated normal tissues. However, in this report, we shall restrict "tumour-bed effect" to mean growth-rate changes which result from treatment of normal tissue only (as when untreated tumours are implanted in pre-treated normal tissues) and shall use "slowed regrowth *in situ*" to describe growth-rate changes which may be observed when the tumour and its supporting normal tissues both receive treatment.

### MATERIALS AND METHODS

The tumour used in these experiments was the rat fibrosarcoma SSB1a, the biological properties of which have been described previously (Howlett *et al.*, 1975). The tumour arose spontaneously in a John's Wistar rat, but was subsequently propagated in Wistar/CFHB hybrid rats, which were the animals used in the experiments described here. The immunological status of this tumour system has not been thoroughly investigated, but the transplantation "take rate" averaged 95% in >1000 transplantations, and no spontaneous regressions were recorded. All tumours were implanted s.c. in the form of 1mm cubes in the mid-dorsal region, just to one side of the mid-line, while the rat was under ether anaesthesia. The incision was closed with a Michel surgical clip which was removed 3 days later. Typically, tumours became palpable 6–10 days after implantation, and were thereafter measured 3 times a week, in 3 mutually perpendicular dimensions, using calipers. Animals were assigned to treatment groups when the geometric mean diameter of the tumour was within the range 7–10 mm. Tumours attached to the skin were discarded. In all experiments, both male and female rats were used, in roughly equal proportions.

*Anaesthesia.*—In all treatment procedures, the rats were anaesthetized by i.p. injection of a neuroleptanalgesic mixture (Hypnorm, Crown Chemical Co.) at a dose level of 1 ml/kg for males, reduced by 8% for females (see Green, 1980). After treatment, recovery was accelerated by administration of a Naloxone antidote (Narcan Neonatal, Winthrop Labs) at a dose level of 0·2 ml/kg.

*X-irradiation.*—When the animals were fully anaesthetized, 2 sutures were passed through the dorsal skin, inferior and superior to the tumour. The rat was placed prone on a jig with an overhead strut and each suture was tied to the strut, thus pulling the tumour and surrounding skin well clear of the vertebrae.

X-irradiation was carried out using a 250kV Marconi X-ray set (filtration: 0·3 mm Cu, 1 mm Al; half-value layer: 1·35 mm Cu). The tumour, supported by the sutures, was placed flush with the collimator and the X-rays were delivered horizontally at a dose rate of 3·75 Gy/min. Animals were irradiated individually and turned through 180° halfway through the irradiation to improve the dose uniformity. The estimated dose variation within the tumour was ⩽4% and the total-body dose, mainly due to internal scatter, was ⩽3%. Each dose group used 6–10 animals.

For irradiation of the tumour bed before transplantation, the procedure was very similar to that described above, but animals were treated before tumour transplantation, so that only the skin and associated normal tissues were irradiated.

After treatment, the perimeter of the treated area was clearly marked by painting with picric acid, and tumours were then implanted within the treated region, within 24 h of treatment. The developing tumours were measured as described previously. Tumours which grew outside the treatment area, or were attached to muscle, were discarded from the study. In each dose group, 6–10 animals were used, and of these about one-third (on average) were discarded because of outside growth or attachment.

*Hyperthermia.*—Hyperthermia treatment was carried out with the tumours rendered hypoxic by mechanical clamping to eliminate blood flow and facilitate uniform heating. This was found (in a previous study) to improve uniformity of the regrowth delay. However, clamping without heating had no effect on tumour growth, and induced no additional regrowth delay.

For these studies, the tumour and surrounding skin was pulled away from the vertebrae and passed through a slot (1 of 6) cut in a plastic tray. The tumour and skin were then firmly clamped between two sections of a plastic clamp, which was tightened with a screw.

When all 6 rats had been positioned below the tray, the tray was inverted so that the rats lay on top in a supine position, with the clamped tumours protruding beneath the slots. The tray was then fitted on to a precision-controlled circulating water-bath (accuracy ±0·05°C) at a pre-set temperature of 43·5°C, with the clamped tumours immersed, but the bodies of the rats well clear of the water.

In a separate study, thermocouple probes were inserted into the tumour and found to stabilize at a temperature 0·2–0·5°C below the temperature of the water-bath, within 2–4 min of immersion. However, it is not possible to exclude the existence of isolated "cold spots" within the tumour.

For each treatment time, 12–20 animals per group were used.

For effects on the tumour bed, clamped dorsal skin was heated before tumour implantation. After heating, the clamps were removed and the perimeter of the treated area marked with picric acid. Within 24 h of treatment, tumour implantation was carried out and the implanted tumour placed close to the centre of the treated region. The tumours were then measured as before, discarding from the study those tumours which grew outside the treated area or were attached to muscle.

For each treatment time, 12–20 animals were used per group and, of these, about one-quarter were discarded from the study.

*Growth-curve analysis.*—Growth curves were plotted and analysed for each tumour individually. This was considered necessary because composite growth curves bias in favour of slow growing tumours (whose host animals are killed later) (see Begg, 1980).

Within the size range 5–20 mm geometric-mean diameter, individual growth curves conformed well to an exponential increase of mean diameter with time; Gompertz retardation becoming apparent only at larger sizes. This was true both for untreated tumours and for tumours regrowing after X-rays or hyperthermia. Tumours recurrent after X-rays or hyperthermia usually followed exponential regrowth patterns.

Growth rates were quantified by fitting the equation

$$\ln [\text{diameter}] = \mu\ [\text{time since treatment}] + \text{constant}$$

to growth data for each individual tumour, using the method of least squares. Then the quantity $\mu$ provides a measure of the rate of growth of each individual tumour and the arithmetic mean $\bar{\mu}$ for a group of tumours provides a measure of the average growth rate for this group.

For untreated (control) tumours, individual growth rates were calculated within the size range of geometric mean diameter 7–20 mm. For tumours recurrent after X-irradiation, the preceding rule was applied, with the proviso that analysis was confined to the monotonic ascending limb of the growth curve (*i.e.* regression phase and "static" phase were excluded from the analysis). For tumours recurring after hyperthermia, a period of transient oedema invariably followed, and analysis was therefore confined to the post-oedema phase of regrowth. Tumour growth was usually analysable only within the size region 10–20 mm mean diameter. Within this region, exponential growth was again observed for the great majority of recurrent tumours.

RESULTS

Fig. 1 shows the relationship between the regrowth time (time to grow to 15 mm geometric-mean diameter) and severity of treatment, for X-ray doses up to 30 Gy and for hyperthermia treatment (43·5°C water-bath) up to 60 min.

As may be seen, the hyperthermia dose–response curve is approximately linear, whilst the X-irradiation dose–response curve has a more complex shape.

Fig. 2 shows the relationship between the mean growth rate ($\bar{\mu}$) defined previously, and severity of treatment, for X-rays and hyperthermia.

For hyperthermia, the growth rates show no simple relationship to severity of treatment, but remain close to the

Fig. 1.—Regrowth delay (time to grow from treatment size to 15 mm mean diameter) for X-irradiation (●——●) and for hyperthermia treatment time at 43·5°C ○---○.

level for untreated tumours within the range of experimental errors.

For X-irradiation, the regrowth rate initially declines with increasing dose, reaching a plateau (above 10 Gy) when

Fig. 2.—Mean specific exponential regrowth rate ($\mu$) *in situ* for tumours regrowing (within the size range 7–20 mm diameter) after X-irradiation (●——●) or hyperthermia (○---○).

Fig. 3.—Mean specific exponential growth rate ($\mu$) for untreated tumours growing in sites previously treated with X-rays (●——●) or hyperthermia (○---○).

Table.—*Summary of* in situ *responses and tumour-bed effects for X-rays and hyperthermia, using the rat fibrosarcoma SSB1a*

| | X-irradiation | | | Hyperthermia | | | |
|---|---|---|---|---|---|---|---|
| | Regrowth *in situ* | | Tumour-bed effect | | Regrowth *in situ* | | Tumour-bed effect |
| Dose (Gy) | Growth delay (days) | Specific growth rate ($\mu$) (ln mm/day) | Specific growth rate ($\mu$) (ln mm/day) | Time at 43·5 °C (min) | Growth delay (days) | Specific growth rate ($\mu$) (ln mm/day) | Specific growth rate ($\mu$) (ln mm/day) |
| 0 | 5·91 ± 0·44 | 0·106 ± 0·003 | 0·106 ± 0·003 | 0 | 5·91 ± 0·44 | 0·106 ± 0·003 | 0·106 ± 0·003 |
| 5 | 5·44 ± 0·34 | 0·079 ± 0·008 | 0·096 ± 0·006 | 10 | 9·03 ± 0·50 | 0·096 ± 0·004 | 0·108 ± 0·005 |
| 10 | 8·99 ± 0·84 | 0·055 ± 0·006 | 0·090 ± 0·017 | 20 | 9·73 ± 0·60 | 0·114 ± 0·005 | 0·102 ± 0·006 |
| 15 | 10·18 ± 0·80 | 0·059 ± 0·008 | 0·075 ± 0·013 | 30 | 13·94 ± 1·16 | 0·128 ± 0·006 | 0·110 ± 0·006 |
| 20 | 11·61 ± 0·90 | 0·059 ± 0·007 | 0·084 ± 0·006 | 45 | 18·54 ± 1·47 | 0·099 ± 0·007 | 0·101 ± 0·004 |
| 25 | 22·50 ± 4·77 | 0·046 ± 0·005 | 0·074 ± 0·004 | 52 | 21·48 ± 1·78 | 0·110 ± 0·014 | |
| 30 | 23·10 ± 6·35 | 0·063 ± 0·007 | 0·083 ± 0·007 | | | | |

*Notes* (1) All means are given ± s.e.

(2) Statistical analysis used the Wilcox–Mann–Whitney *U* test to assess the significance of the difference between mean growth rates for both X-rays and hyperthermia. This analysis showed that:

(a) The mean regrowth rate *in situ* after X-rays (5–30 Gy) is significantly different from the regrowth rate after hyperthermia (10–52 min) ($P < 0·01$).

(b) The corresponding growth rates in tumour-bed effect experiments also differed significantly ($P < 0·01$).

FIG. 4.—Possible consequences of a differential regrowth rate after different forms of treatment. Treatment B produces the lower cell survival but (because of fast regrowth), the shorter growth delay.

the growth rate is about half the corresponding value for untreated tumours.

Fig. 3 shows the relationship of growth rate to severity of treatment for untreated tumours implanted in previously treated normal tissues. As with regrowth *in situ*, for X-irradiation, the growth rate initially declines with treatment, plateauing (above 10 Gy) at a growth rate about three-quarters of that for control tumours. For hyperthermia, the growth rate again simply scatters around the mean for control tumours, with no obvious effect of severity of treatment.

In addition to the graphical presentations in Figs. 1–3, the results of these experiments are summarized in the Table.

### DISCUSSION

The results presented show that, for similar levels of growth delay, tumours regrow more slowly after X-irradiation than after hyperthermia. In addition, tumours implanted in pre-irradiated normal tissue grow more slowly than tumours implanted in pre-heated tissue.

The latter finding agrees with that reported by Urano & Cunningham (1980); *i.e.* a significant tumour-bed effect for X-irradiation but not for hyperthermia. However, these authors compared treatments giving equivalent effects on normal skin (as assessed by skin scoring) rather than equivalent anti-tumour effects, and did not assess regrowth *in situ*.

Slowed regrowth *in situ* after X-irradiation is well recognized (Suit & Shalek, 1963; Thomlinson & Craddock, 1967; Hawkes *et al.*, 1968). Less uniform and more erratic growth patterns have also been reported (Brown & Howes, 1974; Abdelaal *et al.*, 1980) though this was not the case in the present study.

The tumour-bed effect, which may be partly responsible for slowed growth *in situ*, is also well known (Stenstrom *et al.*, 1955; Summers *et al.*, 1964; Hewitt & Blake, 1968; Urano & Suit, 1971) and may result from a reduced capacity of the irradiated tumour bed to provide adequate vascularization for the growing tumour (Thomlinson & Craddock, 1967; Clifton & Jirtle, 1975; Jirtle *et al.*, 1978).

If vascular incapacity is responsible for the tumour-bed effect, it is perhaps surprising that this effect should not occur after hyperthermia, since vascular damage has been reported for hyperthermia treatments within the range reported here (Falk, 1980; Song *et al.*, 1980). However, the relevant factor may be the ability of the treated tissue to respond to tumour demands for neovascularization rather than the functional competence of existing vascular structures. Alternatively, early expression of cellular damage by hyperthermia (*e.g.* intermitotic cell death) could lead to earlier tissue recovery and repopulation after hyperthermia than after radiation, for which damage may remain latent until cell division is provoked by the vascular demands of the developing tumour.

Since the observed regrowth delay depends both upon the number of clonogenic tumour cells surviving treatment and upon the rates at which these surviving cells repopulate, treatment-induced growth changes may have a marked effect on the resultant growth delay. An extreme example is schematically illustrated in Fig. 4. This shows how, in principle, a differential effect on regrowth kinetics could lead to a reversal of the order of effectiveness of the two modalities; *i.e.* the modality with the lesser cell kill but the slower regrowth being judged "more effective" (in terms of growth delay) than the modality giving greater cell kill but rapid regrowth. More generally, a differential effect on regrowth kinetics will lead to a divergence of the growth delay assay from those based on clonogenic cell survival, the modality giving the faster regrowth being systematically undervalued.

Whether or not the effects described in this paper occur during the initial repopulation phase of cells is not known. Even for X-irradiation, the size range over which the tumour-bed effect occurs is by no means clear. Theoretically, it may be expected that the effect would be confined to the "vascular" phase of tumour growth (*i.e.* macroscopic sizes) and this expectation is supported by the study of Hewitt & Blake (1968), who found no effect of tumour-bed irradiation on the latent period for development of tumours from implanted cells to the point of detectability. By contrast, Urano & Suit (1971) and Abdelaal *et al.* (1980) observed a significant prolongation of latency (albeit with a larger end-point) as well as a slowed growth of macroscopic tumours.

Whether the tumour-bed effect is exclusively responsible for slowed regrowth *in situ*, or is only one component, is not known. Theoretically, surviving cells regrowing *in situ* are in a very different environment from cells implanted in an irradiated tumour bed (*e.g.* the pre-existence of tumour blood vessels in the former). However, Hewitt & Blake (1968) demonstrated a tumour-bed effect in sites of tumours irradiated *in situ*, whilst Abdelaal *et al.* (1980) implanted cells both in irradiated (non-tumour-bearing) normal tissue and in the sites of tumours previously cured by irradiation, and observed similar growth slowing in both cases. These results are consistent with slowed regrowth *in situ* being largely or wholly due to a tumour-bed effect. However, Abdelaal *et al.* (1980) also noted a severe non-uniformity for regrowth *in situ* which did not occur for tumours developing in pre-irradiated normal tissue, indicating some differences between the two situations.

In the present series of experiments, growth and regrowth patterns remained reasonably uniform throughout, but the reduction of overall growth rate was appreciably greater for regrowth *in situ* than for tumours implanted into pre-irradiated normal tissues. It seems possible that while the tumour-bed effect makes an important contribution to slowed regrowth *in situ*, additional factors (*e.g.* sublethal damage to tumour cells) may also contribute.

Since changes in growth kinetics are a principal obstacle to the estimation of cell survival *in situ* by back-extrapolation of regrowth curves (see Denekamp, 1980; Wheldon, 1980), the apparent absence of such effects for hyperthermia could make *in situ* estimates of cell survival more feasible for this modality than for others.

It is possible that some of the differences noted here for X-irradiation or hyperthermia may be due to different cells being sensitive or resistant to each of the two modalities (*e.g.* hyperthermia may preferentially kill cells resistant to radiation) so that similar regrowth patterns would not necessarily follow treatments which are iso-effective for cell kill. However, the implications of such a possibility have seldom been considered.

Finally, it should be noted that different kinetic effects of treatment may occur for different cytotoxic drugs, as well as for the treatment modalities reported here (see Stephens & Peacock, 1977; Peacock & Stevens 1978). Evaluation of the magnitude of such treatment-induced kinetic changes is necessary for confident interpretation of regrowth delay experiments, especially where more than one treatment modality is involved.

### REFERENCES

ABDELAAL, A. S., WHELDON, T. E. & CLARKE, B. M. (1980) Perturbation of the growth kinetics of C3H mouse mammary carcinoma by irradiation of tumour and host and by attempted pre-immunization of host. *Br. J. Cancer*, **41**, 567.

BEGG, A. C. (1980) Analysis of growth delay data: potential pitfalls. *Br. J. Cancer*, **41**, (Supp. IV), 93.

BREUR, K. R. (1966) Growth rate and radiosensitivity of human tumours. *Eur. J. Cancer*, **2**, 157.

BROWN, J. M. & HOWES, A. G. (1974) Comparison of tumour growth delay with cell survival. *Br. J. Radiol.*, **47**, 509.

CLIFTON, K. H. & JIRTLE, R. (1975) Mammary carcinoma population growth in pre-irradiated and unirradiated transplant sites. *Radiology*, **117** 459.

DENEKAMP, J. (1980) Is any single *in situ* assay of tumour response adequate? *Br. J. Cancer*, **41**, (Suppl. IV), 56.

FALK, P. (1980) The vascular pattern of the spontaneous C3H mouse mammary carcinoma and its significance in radiation response and in hyperthermia. *Eur. J. Cancer*, **16**, 203.

GREEN, C. J. (1979) *Animal Anaesthesia*. London: Laboratory Animals Ltd.

HAWKES, M. J., HILL, R. P. LINDOP, P. J., ELLIS, R. E. & ROTBLAT, J. (1968) The response of C3H mammary tumours to irradiation in single and fractionated doses. *Br. J. Radiol.*, **41**, 134.

HEWITT, H. B. & BLAKE, E. R. (1968) The growth of transplanted tumours in pre-irradiated sites. *Br. J. Cancer*, **22**, 808.

HOWLETT, J. F., THOMLINSON, R. H. & ALPER, T. (1975) A marked dependence of the comparative effectiveness of neutrons on tumour line and its implications for clinical trials. *Br. J. Radiol.*, **48**, 40.

JIRTLE, R., RANKIN, J. H. G. & CLIFTON, K. H. (1978) Effects of X-irradiation on tumour blood flow and vascular response to drugs. *Br. J. Cancer*, **37**, 1033.

MCNALLY, N. J. (1974) Tumour growth and cell survival *in situ*. *Br. J. Radiol.*, **47**, 510.

PEACOCK, J. H. & STEPHENS, T. C. (1978) Influence of anaesthetics on tumour cell kill and repopulation in B16 melanoma treated with melphalan. *Br. J. Cancer*, **38**, 725.

SONG, C. W. KANG, M. S., RHEE, J. G. & LEVITT, S. H. (1980) Vascular damage and delayed cell death in tumours after hyperthermia. *Br. J. Cancer*, **41**, 309.

STENSTROM, K. W., VERMUND, H., MOSSER, D. G. & MARVIN, J. F. (1955) Effects of röntgen radiation on the tumour bed. *Radiat. Res.*, **2**, 180.

STEPHENS, T. C. & PEACOCK, J. H. (1977) Tumour volume response, initial cell kill and cellular repopulation in B16 melanoma treated with cyclophosphamide and CCNU. *Br. J. Cancer*, **36**, 313.

SUIT, H. D. & SHALEK, R. J. (1963) Response of anoxic C3H mouse mammary carcinoma isotransplants to X-irradiation. *J. Natl Cancer Inst.*, **31**, 479.

SUMMERS, W. C., CLIFTON, K. H. & VERMUND, H. (1964) X-irradiation of the tumour bed. I. A study of the indirect actions of radiation on transplantable tumours. *Radiology*, **82**, 691.

THOMLINSON, R. H. & CRADDOCK, E. A. (1967) The gross response of an experimental tumour to single doses of X-rays. *Br. J. Cancer*, **21**, 108.

URANO, M. & SUIT, H. D. (1971) Experimental evaluation of tumour bed effect for C3H mouse mammary carcinoma and for C3H mouse fibrosarcoma. *Radiat. Res.*, **45**, 41.

URANO, M. & CUNNINGHAM, M. (1980) Insignificant tumour bed effect after pre-transplantation hyperthermia. *Cancer Res.*, **40**, 26.

WHELDON, T. E. (1980) Can dose–survival parameters be deduced from *in situ* assays? *Br. J. Cancer*, **41** (Suppl. IV), 79.


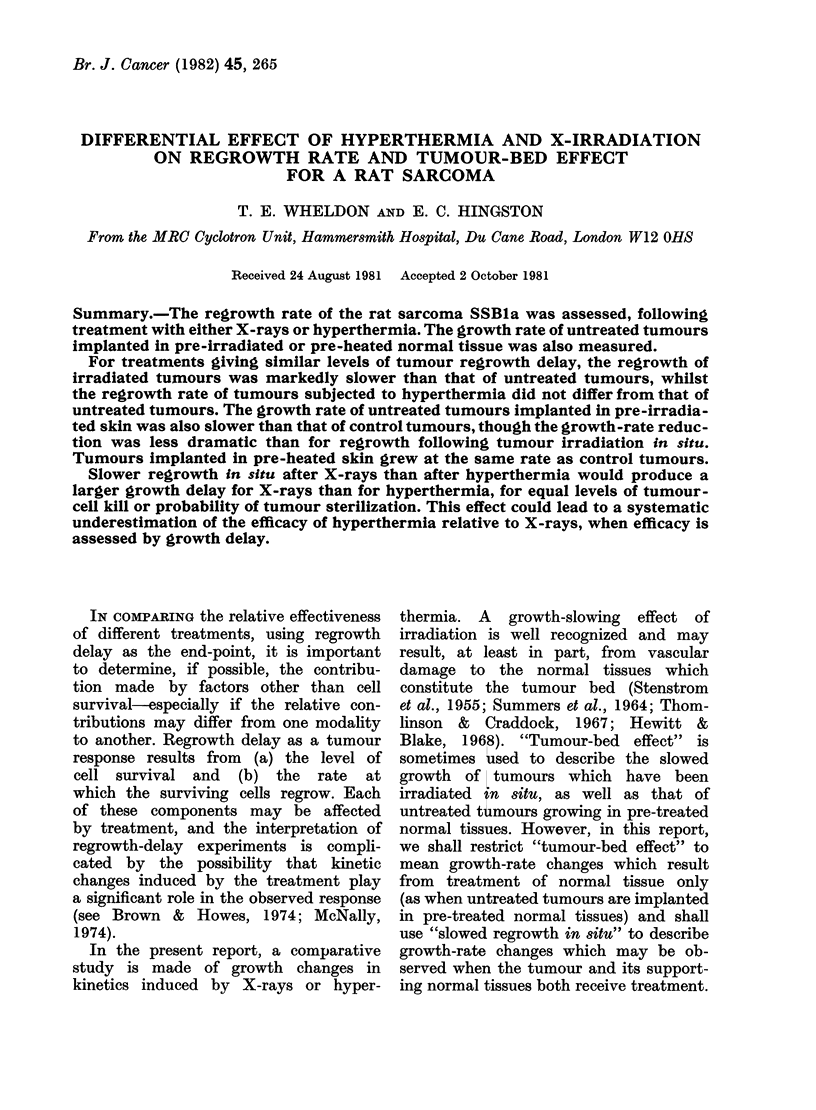

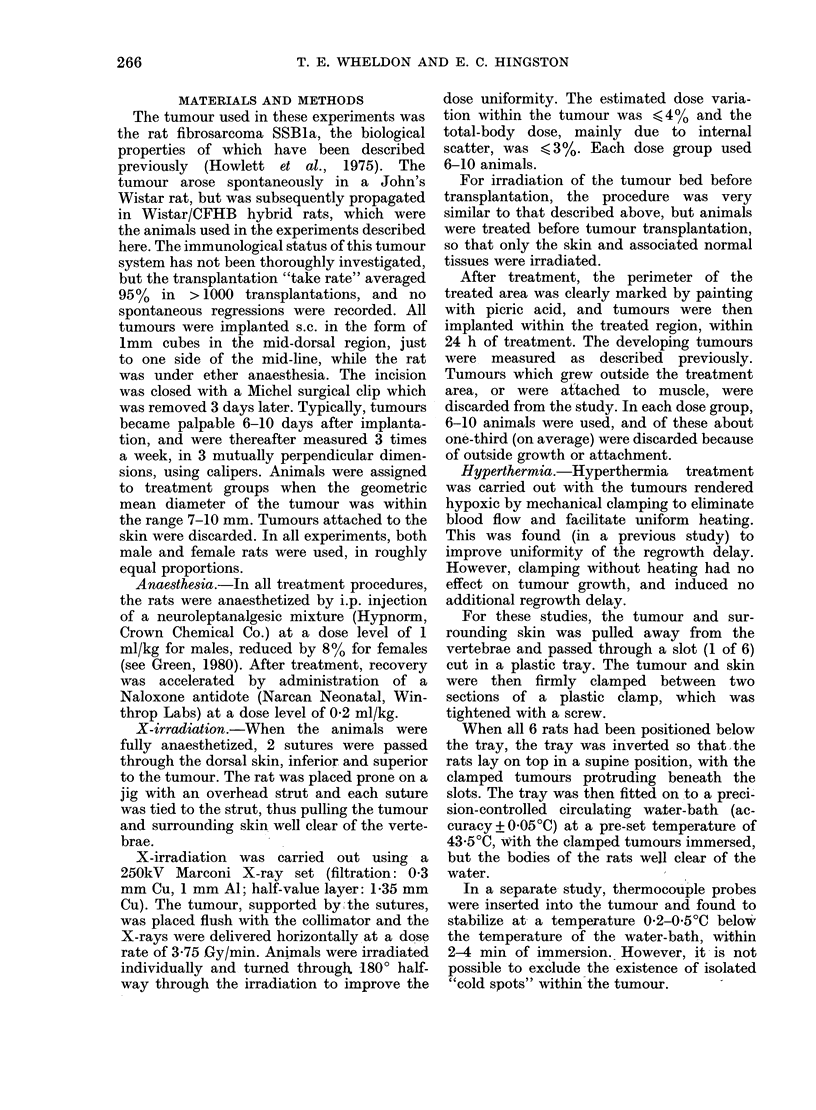

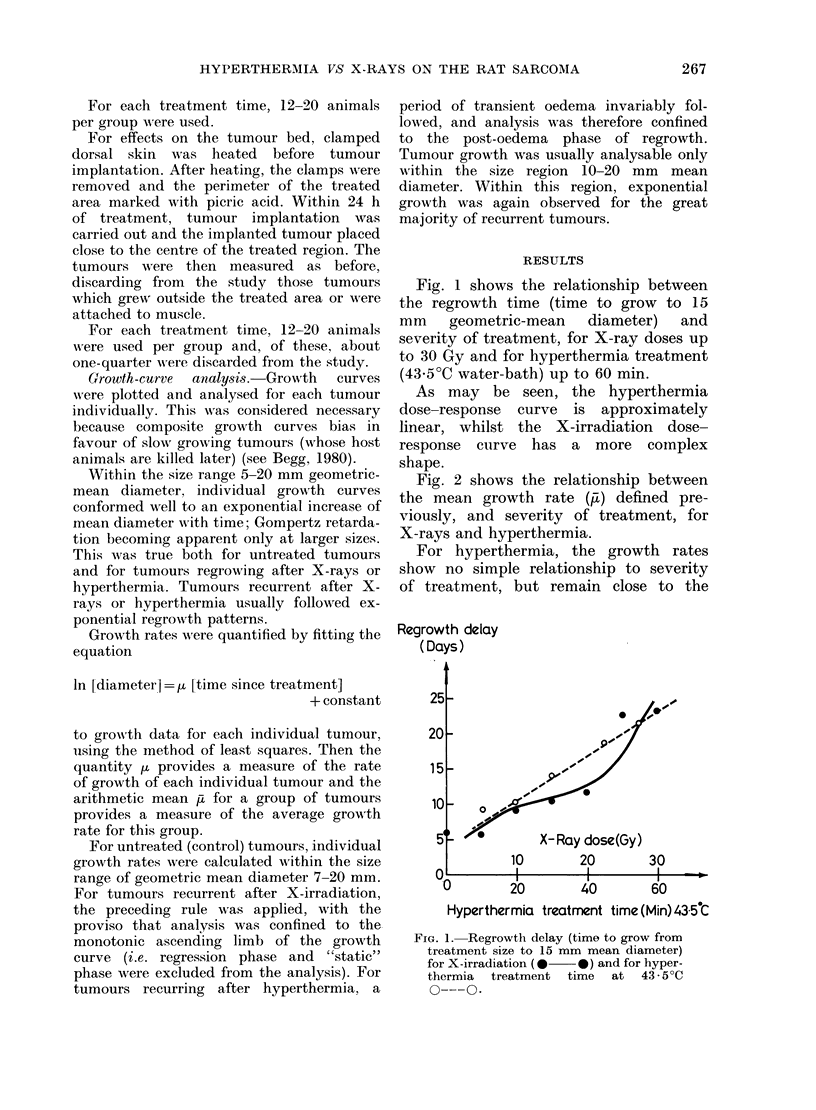

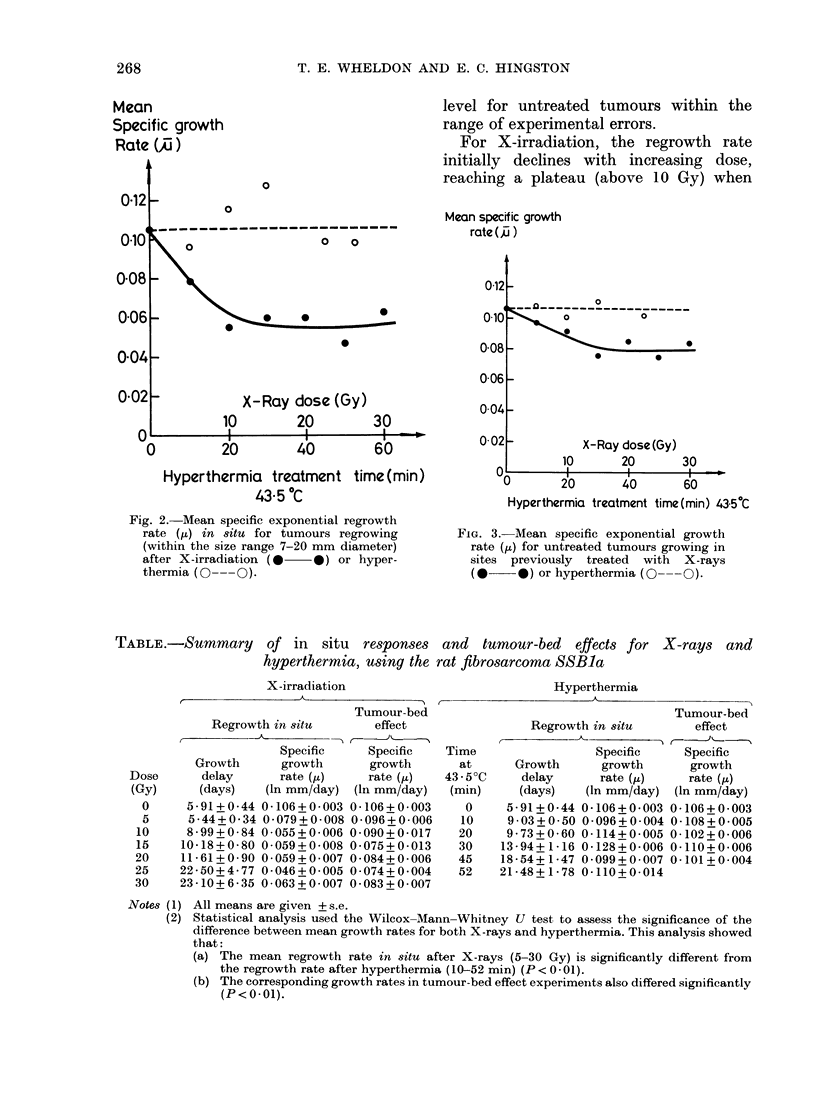

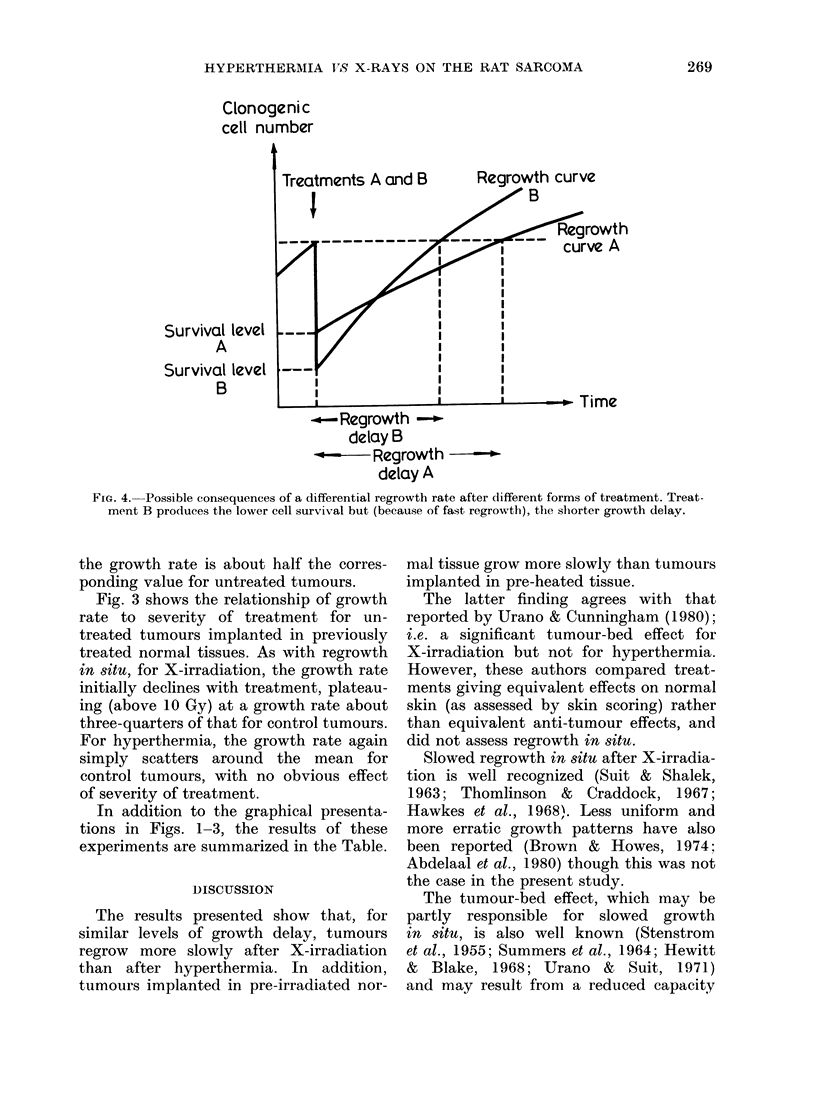

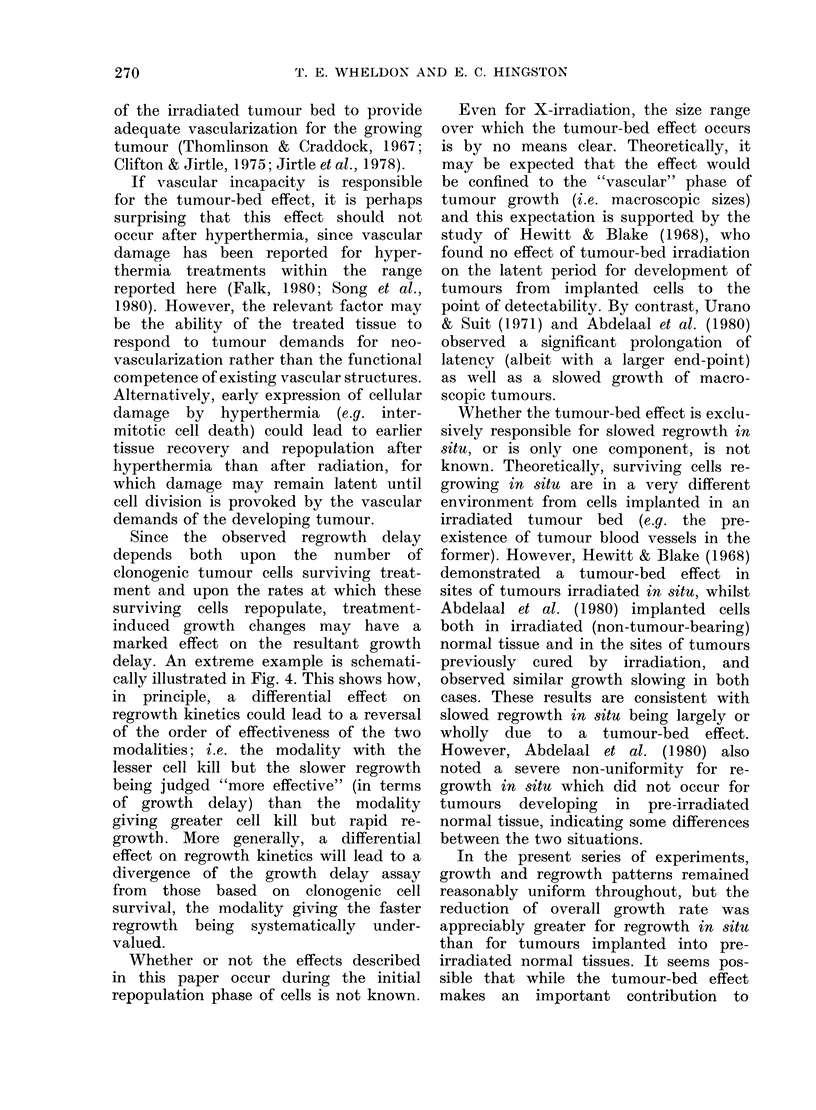

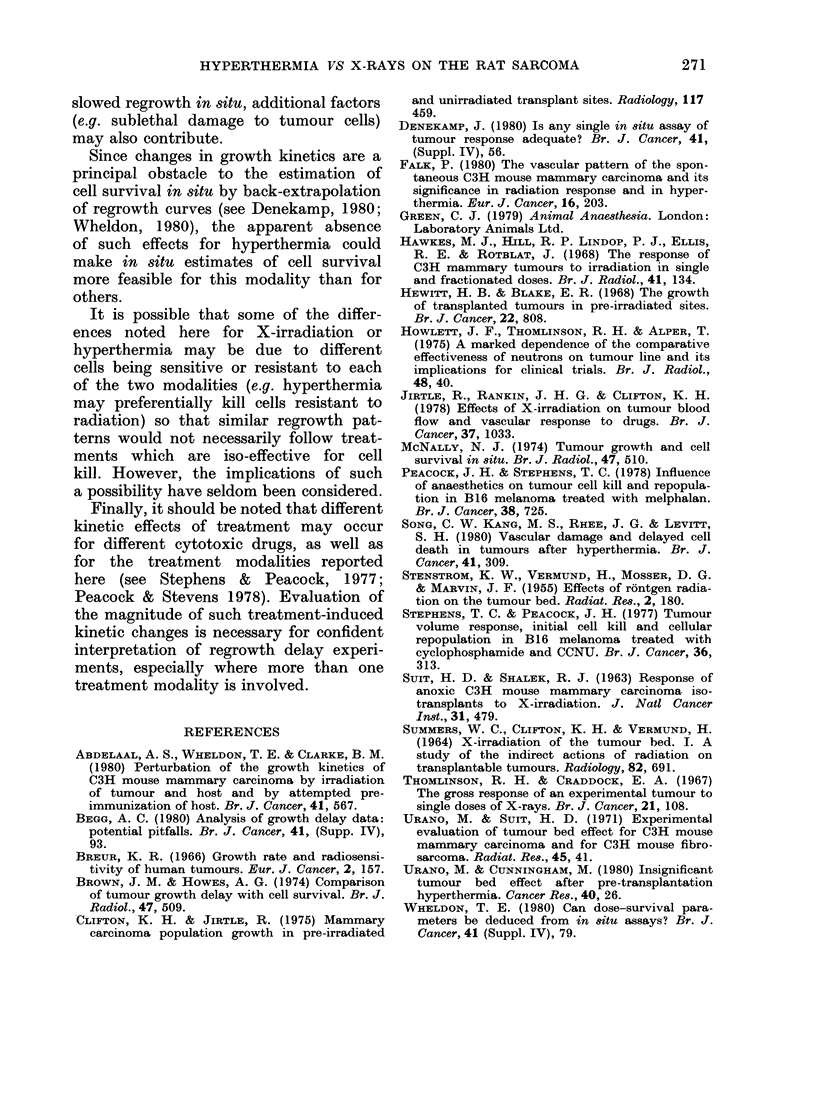


## References

[OCR_00783] Abdelaal A. S., Wheldon T. E., Clarke B. M. (1980). Perturbation of the growth kinetics of C3H mouse mammary carcinoma by irradiation of tumour and host and by attempted pre-immunization of host.. Br J Cancer.

[OCR_00795] Breur K. (1966). Growth rate and radiosensitivity of human tumours. I. Growth rate of human tumours.. Eur J Cancer.

[OCR_00798] Brown J. M., Howes A. E. (1974). Letter: Comparison of tumour growth delay with cell survival.. Br J Radiol.

[OCR_00803] Clifton K. H., Jirtle R. (1975). Mammary carcinoma cell population growth in preirradiated and unirradiated transplant sites. Viable tumor growth, vascularity, and the tumor-bed effect.. Radiology.

[OCR_00815] Falk P. (1980). The vascular pattern of the spontaneous C3H mouse mammary carcinoma and its significance in radiation response and in hyperthermia.. Eur J Cancer.

[OCR_00825] Hawkes M. J., Hill R. P., Lindp P. J., Ellis R. E., Rotblat J. R. (1968). The response of C3H mammary tumours to irradiation in single and fractionated doses.. Br J Radiol.

[OCR_00831] Hewitt H. B., Blake E. R. (1968). The growth of transplanted murine tumours in pre-irradiated sites.. Br J Cancer.

[OCR_00836] Howlett J. F., Thomlinson R. H., Alper T. (1975). A marked dependence of the comparative effectiveness of neutrons on tumour line, and its implications for clinical trials.. Br J Radiol.

[OCR_00843] Jirtle R., Rankin J. H., Clifton K. H. (1978). Effect of x-irradiation of tumour bed on tumour blood flow and vascular response to drugs.. Br J Cancer.

[OCR_00849] McNally N. J. (1974). Letter: Tumour growth delay and cell survival "in situ".. Br J Radiol.

[OCR_00853] Peacock J. H., Stephens T. C. (1978). Influence of anaesthetics on tumour-cell kill and repopulation in B16 melanoma treated with melphalan.. Br J Cancer.

[OCR_00865] STENSTROM K. W., VERMUND H., MOSSER D. G., MARVIN J. F. (1955). Effects of roentgen irradiation on the tumor bed. I. The inhibiting action of local pretransplantation roentgen irradiation (1500 r alpha) on the growth of mouse mammary carcinoma.. Radiat Res.

[OCR_00877] SUIT H. D., SHALEK R. J. (1963). RESPONSE OF ANOXIC C3H MOUSE MAMMARY CARCINOMA ISOTRANSPLANTS (1-25 MM3) TO X IRRADIATION.. J Natl Cancer Inst.

[OCR_00883] SUMMERS W. C., CLIFTON K. H., VERMUND H. (1964). X-IRRADIATION OF THE TUMOR BED. I. A STUDY OF THE INDIRECT ACTIONS OF RADIATION ON TRANSPLANTABLE TUMORS.. Radiology.

[OCR_00859] Song C. W., Kang M. S., Rhee J. G., Levitt S. H. (1980). Vascular damage and delayed cell death in tumours after hyperthermia.. Br J Cancer.

[OCR_00870] Stephens T. C., Peacock J. H. (1977). Tumour volume response, initial cell kill and cellular repopulation in B16 melanoma treated with cyclophosphamide and 1-(2-chloroethyl)-3-cyclohexyl-1-nitrosourea.. Br J Cancer.

[OCR_00889] Thomlinson R. H., Craddock E. A. (1967). The gross response of an experimental tumour to single doses of x-rays.. Br J Cancer.

[OCR_00900] Urano M., Cunningham M. (1980). Insignificant tumor bed effect after pretransplantation hyperthermia.. Cancer Res.

[OCR_00894] Urano M., Suit H. D. (1971). Experimental evaluation of tumor bed effect for C3H mouse mammary carcinoma and for C3H mouse fibrosarcoma.. Radiat Res.

[OCR_00905] Wheldon T. E. (1980). Can dose-survival parameters be deduced from in situ assays?. Br J Cancer Suppl.

